# Immediate Kangaroo Mother Care for Sick Newborns: Quality Improvement Implementation and Feasibility Study

**DOI:** 10.2196/78207

**Published:** 2025-09-26

**Authors:** Belén Fernández-Monteaguado, Ana Peña-Moreno, Inés Ramírez-de Andrés, Patricia Barbero-Casado, Eduardo Zarzuela-NÚñez, Beatriz Bellón-Vaquerizo, Cristina Martin Arriscado-Arroba, María Teresa Moral-Pumarega, Salvador Piris-Borregas, Carmen Rosa Pallás-Alonso

**Affiliations:** 1 Neonatology Department Hospital Universitario 12 De Octubre Madrid Spain; 2 Obstetrician Department Hospital Universitario 12 de Octubre Madrid Spain; 3 Obstetric Reanimation Hospital Universitario 12 de Octubre Madrid Spain; 4 Research institute Hospital Universitario 12 de Octubre Madrid Spain; 5 Maternal and Child Health and Development Research Network (RICORS-SAMID Network) Madrid Spain

**Keywords:** kangaroo mother care method, premature newborns, respiratory distress syndrome, newborn, congenital abnormalities, heart defects, congenital

## Abstract

**Background:**

A systematic separation of late preterm and/or sick newborns from their mothers and families continues to occur in most neonatal units around the world.

**Objective:**

The aim of this study was to implement immediate kangaroo mother care for sick newborns from birth.

**Methods:**

This study comprised preterm newborns at ≥34 weeks of gestational age and weighing ≥1800 g, term newborns with noninvasive ventilation, and newborns at ≥34 weeks of gestational age and weighing ≥1800 g with congenital anomalies that did not require urgent medical or surgical attention. Newborns were excluded in cases of advanced resuscitation or at medical discretion. The newborns in this study were maintained skin-to-skin for 120 minutes, preferably with the mother, and then transferred skin-to-skin to their destination unit.

**Results:**

In this study, 60 newborns were included. The median time for immediate kangaroo mother care was 120 minutes for all newborns; 100% of the time was spent with the mother, even when respiratory support was required. Immediate kangaroo mother care was interrupted for 13 of the 60 newborns, and this was more frequent in newborns with heart disease (9/13, 69%), with the main cause being the neonatologist's concern. No causes of separation were related to maternal issues, hypoglycemia, or temperature instability, and no incidents such as hypothermia were observed during kangaroo mother care, either at birth or during transport.

**Conclusions:**

Kangaroo mother care during the first 120 minutes of life in late premature or sick newborns is a safe and feasible practice.

## Introduction

An increasing body of evidence supports maintaining the nuclear family unit at birth by promoting early newborn stabilization, prolonged uninterrupted immediate kangaroo mother care (iKMC) as soon as possible after birth, and initiating breastfeeding on the mother's bare chest, which is known as iKMC [1-3]. iKMC for preterm newborns has been associated with reduced mortality, nosocomial infections, and hypothermia in those weighing less than 1500 g [4]. It also improves breastfeeding rates [5,6], reduces parental stress and health care costs [7], and enhances long-term health outcomes [8]. Hence, the World Health Organization (WHO) recommends iKMC for neonates born after 28 weeks of gestation [9].

There is evidence regarding the feasibility and safety of iKMC in very premature newborns [10]. Nevertheless, there is a lack of clinical protocols or experiences explaining how to implement iKMC in other groups of ill newborns, particularly, late premature newborns, those requiring noninvasive respiratory support, or those with prenatal diagnoses. In many cases, there is very little evidence for these groups. This study aims to evaluate the feasibility and safety of iKMC at birth for sick newborns, thereby bringing the benefits of iKMC to a greater number of sick newborns and their families.

## Methods

### Study Design

This is a quality improvement study. The inclusion criteria comprised 3 gestational groups: (1) preterm newborns between ≥34+0 and <37+0 weeks of gestational age (GA) and weighing ≥1800 g with or without respiratory distress requiring or not noninvasive ventilation (group 1); (2) term neonates ≥37+0 weeks of GA with respiratory distress (group 2); and (3) term newborns ≥37+0 weeks of GA and preterm newborns ≥34 weeks of GA, weighing ≥1800 g, and having a prenatal congenital anomaly not requiring immediate medical or surgical intervention (group 3). The exclusion criteria were neonates needing intubation, cardiac massage, advanced resuscitation, noninvasive ventilation with FiO₂ (fraction of inspired oxygen) >40%, or with heart rate <100 beats per minute, as well as those excluded based on clinical judgment by the attending team. The iKMC protocol established a standardized duration of 120 minutes, irrespective of the delivery mode and was conducted in either the labor/recovery unit or the obstetric resuscitation area. Continuation of iKMC was permitted in the assigned maternal or neonatal unit depending on clinical stability. All training sessions were multidisciplinary, attended by neonatologists, resident doctors, neonatal nurses, midwives, and anesthetists.

A neonatologist or neonatal nurse was present at the initiation of skin-to-skin contact. In delivery rooms, newborns could be assessed either directly or via telemetry. Monitoring with pulse oximetry and temperature or glucose measurement was recommended, as appropriate. In cases of asymptomatic early mild hypoglycemia, breastfeeding during iKMC was encouraged to avoid mother-newborn separation. For cesarean deliveries, the physical presence of both a neonatologist and a neonatal nurse was mandatory. In all cases, the umbilical cord was cut after 1 minute. The temperature was measured immediately after birth and upon arrival at the neonatal intensive care unit. If at any time the temperature was below 36 °C, iKMC was interrupted. When the temperature was between 36 °C and 36.5 °C, additional measures were initiated, such as preheated cloths or increasing the maternal temperature with air convection blankets. Ideally, transport to the destination unit was performed with the mother using a bed or a wheelchair depending on her physical condition at the time. If the mother was not available, iKMC transport with the father or the other authorized caregiver was encouraged. A wheelchair was used for this purpose.

The primary outcome was the duration of uninterrupted iKMC (in minutes). Additional variables included patient demographics, the individual providing the care, need for respiratory support, and its discontinuation during iKMC. A formal sample size calculation was not conducted; however, a target of 50 patients was deemed sufficient to assess feasibility. Ultimately, 60 patients were included. Quantitative variables were summarized using mean and standard deviation or median (p50) and interquartile range (p25-p75), depending on the distribution assessed by the Shapiro-Wilk test. Categorical variables were reported as absolute counts and percentages. Group comparisons were performed using analysis of variance, Kruskal-Wallis, Mann-Whitney *U* test, 2-sided Student *t* test, chi-square, or Fisher exact test, as appropriate. Bonferroni adjustment was applied for comparisons among the 3 inclusion groups. All analyses were conducted using SAS software (version 9.4, SAS Institute), with statistical significance set at *P*≤.05 and 95% CI.

### Ethical Considerations

This study is a subproject of a larger project called “zero separation.” The larger zero separation project was submitted to the research ethics committee with all the subprojects. The ethics committee of the 12 de Octubre University Hospital approved the global project (approval 25/542). All families, including mothers and fathers, were informed verbally. No informed consent was required according to the recommendations of the ethics committee, and participants could opt out anytime. Data were anonymized, and no compensation was given to any participant.

## Results

Between January and August 2024, 89 eligible newborns were admitted to the unit. Of these, 29 (33%) were excluded: 52% (15/29) were excluded due to respiratory distress with FiO₂>40%, 35% (10/29) due to maternal factors, and 14% (4/29) for reasons such as advanced resuscitation or care overload. The final sample comprised 60 newborns.

The characteristics of the included newborns are shown in Table 1. Significant differences were observed among groups 1, 2, and 3 in the gestational weeks (median 35+0 [IQR 34+2 to 36+0] weeks vs 40+2 [IQR 39+2 to 40+6] weeks vs 40+0 [IQR 38+5 to 40+4] weeks, respectively; *P*<.01) and birth weight (2350 [IQR 2080-2940] g vs 3290 [IQR 3130-3660] g vs 3230 [IQR 2970-3550] g, respectively; *P*<.01). Among the different congenital anomalies, 60% (14/24) were congenital heart diseases such as Tetralogy of Fallot or aortic coarctation, 20% (4/24) were neurological problems, and 10% (3/24) were other anomalies.

The characteristics of iKMC are presented in Table 2. The median duration was 120 minutes for all newborns, with no differences among the groups. All newborns received iKMC with their mothers. Notably, newborns in the respiratory distress group (group 2) more frequently required continuous positive airway pressure (CPAP) than groups 1 and 3 during iKMC (17/19, 90% vs 6/17, 35% vs 1/24, 4%, respectively; *P*<.01) and were more frequently transported in iKMC (16/19, 84% vs 8/17, 47% vs 10/24, 42%, respectively; *P*=.01).

**Table 1:** General characteristics of the sample of newborns recruited based on the inclusion criteria.

Variables		Late preterm newborns (n=17)	Term newborns with respiratory distress (n=19)	Newborns with congenital pathology (n=24)	*P* value	
Gestational age (weeks), median (IQR)		35+0 (34+2 to 36+0)	40+2 (39+2 to 40+6)	40+0 (38+5 to 40+4)	<.01	
Weight (g), median (IQR)		2350 (2080 to 2940)	3290 (3130 to 3660)	3230 (2970 to 3550)	<.01	
Sex (male), n (%)		8 (47)	11 (58)	13 (54)	.80	
Twin pregnancy (yes), n (%)		3 (18)	0 (0)	1 (4)	.08	
Type of delivery (eutocic), n (%)		13 (77)	13 (68)	17 (71)	.55	
Intrauterine growth retardation, n (%)		1 (6)	1 (5)	0 (0)	.50	
Need for resuscitation with positive pressure ventilation, n (%)		3 (18)	5 (26)	1 (4)	.12	
**Shift in which immediate kangaroo mother care was performed, n (%)**						.22
	Morning	9 (53)	7 (37)	5 (21)		
	Afternoon	2 (12)	6 (32)	7 (29)		
	Night	6 (35)	6 (32)	12 (50)		

**Table 2:** Characteristics of the immediate kangaroo mother care in the contact protocol patients.

Variables			Late preterm newborns (n=17)		Term newborns with respiratory distress (n=19)		Newborns with congenital pathology (n=24)		*P* value
Median (IQR) time (min) of uninterrupted kangaroo care			120 (120-120)		120 (120-120)		120 (30-120)		.23
With whom kangarooing was performed (with mother), n (%)			17 (100)		19 (100)		24 (100)		>.99
**CPAP^a^ in kangaroo care (yes), n (%)**			6 (35)		17 (90)		1 (4)		<.01
	Possibility to remove CPAP during kangaroo care	0 (0)		4 (24)		0 (0)		.37	
	Possibility to remove CPAP after 120 min of kangaroo care	0 (0)		5 (29)		0 (0)		.27	
	CPAP maintenance for 120 min and subsequent admission	5 (83)		6 (35)		0 (0)		.28	
	Need to discontinue kangaroo care due to deterioration	1 (17)		2 (12)		1 (100)		.07	
Performance of transport in kangaroo care (yes), n (%)			8 (47)		16 (84)		10 (42)		.01
With whom kangaroo transport was performed (with mother), n (%)			8 (100)		13 (81)		8 (80)		.40
Kangaroo transport incidents (yes), n (%)			0 (0)		0 (0)		0 (0)		>.99

^a^CPAP: continuous positive airway pressure.

Table 3 summarizes the characteristics of iKMC among protocol participants. Among those with interrupted kangaroo care, the median duration was 20 (IQR 20-30) minutes. Early separation occurred more often in newborns with prenatal diagnoses of congenital anomalies (9/24, 69% vs 2/17,15% vs 2/19,15%, respectively; *P*=.02), although the median iKMC duration did not differ significantly between the groups.

Temperature instability and hypoglycemia were not reported as the causes of separation. Newborns who experienced iKMC interruption were less likely to be transported in iKMC (3/13, 23% vs 31/47, 66%, respectively; *P*<.01). No significant differences in interruption rates were observed across work shifts (morning: 3/13, 23%; afternoon: 3/13, 23%; night: 7/13, 54%; *P*=.47). Interestingly, newborns requiring positive pressure ventilation at birth (n=9) were less likely to have iKMC interrupted (2/9, 22% vs 7/9, 78%, respectively; *P*=.02). Some details related with the characteristics of breastfeeding during iKMC and at discharge were also studied, which are shown in Table 4.

**Table 3:** Characteristics of kangaroo care interruption in the contact protocol.

Variables			Late preterm newborns (n=17)		Term newborns with respiratory distress (n=19)		Newborns with congenital pathology (n=24)		*P* value	
Kangaroo care interruption at some point (yes), n (%)			2 (15)		2 (15)		9 (69)		.02	
Median (IQR) time (min) to interruption			35 (30-40)		17.5 (15-20)		20 (20-30)		.17	
**Reason for separation, n (%)**										.09
	Increase in respiratory distress of the newborn	1 (50)		1 (50)		2 (22)				
	Obstetrics complications of the mother	0 (0)		0 (0)		0 (0)				
	Reason not referred/concern of neonatologist	0 (0)		1 (50)		6 (67)				
	Other reasons	1 (50)		0 (0)		1 (11)				

**Table 4:** Characteristics of breastfeeding during kangaroo care and at discharge.

Variables	Late preterm newborns (n=17), n (%)	Term newborns with respiratory distress (n=19), n (%)	Newborns with congenital pathology (n=24), n (%)	*P* value
Breastfeeding in kangaroo care achieved	5 (29)	5 (26)	8 (33)	.88
How to get colostrum (% attachment to the breast)	17 (100)	19 (100)	24 (100)	>.99
Breastfeeding at discharge	17 (100)	17 (90)	23 (96)	.34
Exclusive breastfeeding at discharge	7 (41)	10 (53)	10 (4)	.76

## Discussion

This study represents the first experience published in Europe demonstrating the feasibility and security of iKMC for sick newborns and late premature newborns. Nearly 80% (47/60) of the newborns remained in uninterrupted iKMC for 120 minutes, with 100% of that time spent skin-to-skin with their mothers, even among those requiring positive pressure ventilation or respiratory support. No complications occurred during the process or during skin-to-skin transport.

No studies have been found with which to compare the outcomes of iKMC in patients with prenatally detected congenital anomalies. Walsh et al [11] evaluated singletons born vaginally at 35-36 6/7 weeks gestation who were randomized to be initiated with iKMC with a reported time of immediate kangaroo care of 60 minutes. Our median uninterrupted iKMC duration of 120 minutes is notably longer, likely reflecting heightened staff awareness and targeted training. iKMC was interrupted in 22% (13/60) of the cases, predominantly due to worsening respiratory status (4/13, 31%). No interruptions were related to maternal issues, hypoglycemia, or temperature instability, consistent with prior literature [12,13]. This underscores the role of health care providers, sometimes unintentionally, as the main cause of mother-newborn separation.

Technical or logistical issues accounted for only 2 interruptions. No significant differences in iKMC duration or interruption rates were observed across different shifts or patient subgroups. Numerous studies support the safety and benefits of KMC for newborns requiring respiratory support from birth [13,14]. However, only one prior study comprising premature newborns, including late premature ones, demonstrated safe respiratory stabilization by using noninvasive ventilation during skin-to-skin care, achieving 40.6% stabilization in kangaroo care [13]. In our study, this rate increased to 79% (47/60), with 38% (9/24) of the patients discontinuing CPAP during KMC, thereby reducing admissions. Differences in GAs between studies should be noted.

Notably, newborns requiring respiratory support were more frequently transported skin-to-skin, confirming the feasibility and safety of this method. This study extends existing evidence to preterm and sick newborns. Regarding breastfeeding, iKMC is known to promote early latch and initiation in healthy term newborns [13,15]. Our findings extend this to preterm and sick newborns, with 30% (18/60) achieving early colostrum latch and a greater breastfeeding rate at discharge (57/60, 95%), including exclusive breastfeeding in half of the cohort.

This study is the first to detail the way to apply implementation of iKMC in preterm and sick newborns, achieving greater recruitment and applicability. A limitation is that our study was conducted in a unit with extensive staff training and strong commitment to iKMC, which may limit generalizability without similar institutional support. Indeed, our unit has previously published its experience regarding the implementation of iKMC in preterm newborns born between 28 and 34 weeks of gestation [10].

In conclusion, iKMC with uninterrupted skin-to-skin contact and transport during the first 120 minutes of life in late preterm and sick newborns is safe and feasible. Newborns that required noninvasive ventilation with FiO_2_ <40% could be initiated with iKMC. Early mother-newborn contact also improves breastfeeding initiation. This study represents a further step toward zero separation—a goal strongly desired by parents of hospitalized newborns and actively advocated by the Global Foundation for the Care of the Newborn Infant. To date, most scientific literature has focused primarily on very premature newborns. However, extending the practice of iKMC to newborns with higher GAs has the potential to benefit a much larger number of families by preventing unnecessary separation.

## Abbreviations

CPAP: continuous positive airway pressure
FiO2: fraction of inspired oxygen
GA: gestational age
iKMC: immediate kangaroo mother care
WHO: World Health Organization
